# Combating Fraudulent Participation in Urban American Indian and Alaska Native Virtual Health Research: Protocol for Increasing Data Integrity in Online Research (PRIOR)

**DOI:** 10.2196/52281

**Published:** 2024-06-13

**Authors:** Nicole D Reed, Sheana Bull, Umit Shrestha, Michelle Sarche, Carol E Kaufman

**Affiliations:** 1 Centers for American Indian and Alaska Native Health Colorado School of Public Health University of Colorado Anschutz Medical Campus Aurora, CO United States; 2 Community and Behavioral Health Colorado School of Public Health University of Colorado Anschutz Medical Campus Aurora, CO United States; 3 Colorado School of Public Health Colorado State University Fort Collins, CO United States

**Keywords:** fraudulent survey participation, online survey research, American Indian and Alaska Native, data integrity, health research, research trust, online survey, case study, randomized control trial, RCT, social media, recruitment, young women, women, American Indian, Native Americans, Native American, fraudulent, data privacy

## Abstract

**Background:**

While the advantages of using the internet and social media for research recruitment are well documented, the evolving online environment also enhances motivations for misrepresentation to receive incentives or to “troll” research studies. Such fraudulent assaults can compromise data integrity, with substantial losses in project time; money; and especially for vulnerable populations, research trust. With the rapid advent of new technology and ever-evolving social media platforms, it has become easier for misrepresentation to occur within online data collection. This perpetuation can occur by bots or individuals with malintent, but careful planning can help aid in filtering out fraudulent data.

**Objective:**

Using an example with urban American Indian and Alaska Native young women, this paper aims to describe PRIOR (Protocol for Increasing Data Integrity in Online Research), which is a 2-step integration protocol for combating fraudulent participation in online survey research.

**Methods:**

From February 2019 to August 2020, we recruited participants for formative research preparatory to an online randomized control trial of a preconceptual health program. First, we described our initial protocol for preventing fraudulent participation, which proved to be unsuccessful. Then, we described modifications we made in May 2020 to improve the protocol performance and the creation of PRIOR. Changes included transferring data collection platforms, collecting embedded geospatial variables, enabling timing features within the screening survey, creating URL links for each method or platform of data collection, and manually confirming potentially eligible participants’ identifying information.

**Results:**

Before the implementation of PRIOR, the project experienced substantial fraudulent attempts at study enrollment, with less than 1% (n=6) of 1300 screened participants being identified as truly eligible. With the modified protocol, of the 461 individuals who completed a screening survey, 381 did not meet the eligibility criteria assessed on the survey. Of the 80 that did, 25 (31%) were identified as ineligible via PRIOR. A total of 55 (69%) were identified as eligible and verified in the protocol and were enrolled in the formative study.

**Conclusions:**

Fraudulent surveys compromise study integrity, validity of the data, and trust among participant populations. They also deplete scarce research resources including respondent compensation and personnel time. Our approach of PRIOR to prevent online misrepresentation in data was successful. This paper reviews key elements regarding fraudulent data participation in online research and demonstrates why enhanced protocols to prevent fraudulent data collection are crucial for building trust with vulnerable populations.

**Trial Registration:**

ClinicalTrials.gov NCT04376346; https://www.clinicaltrials.gov/study/NCT04376346

**International Registered Report Identifier (IRRID):**

DERR1-10.2196/52281

## Introduction

Research related to health, health behavior, and risk and protective factors that influence health behavior has proliferated online since the late 20th and early 21st century. In an era dubbed “Web 1.0,” content on the world wide web or internet was largely static [1], promoting material that was unidirectional on a website or landing page. Researchers interested in using the internet to recruit participants were at the vanguard of efforts that focused on documenting behaviors and risk and protective factors such as smoking [2], physical activity [3], sexual health [4], reproductive health [5], substance use [6], and mental health [7]. There are also multiple examples of efforts using the internet to recruit participants in intervention research to facilitate improvements in health behavior and factors that influence health behavior [8-11].

In the earliest examples of internet-based research recruitment, researchers noted the advantages of the medium reflected in the ability to increase recruitment efficiency, with the promise of enrolling larger samples in a shorter time frame [12]. Additionally, researchers noted that the internet offered opportunities to reach hidden, marginalized, or stigmatized groups [13], such as men who have sex with men at elevated risk for HIV [14] and substance users [15]. The rapid advent of a “Web 2.0” environment online [16], where users could engage with content on websites and generate their own content, was followed quickly by the earliest social media platforms, such as MySpace and Facebook, in the first decade of the 21st century [17], which allowed users to share content with each other. Efforts to use these platforms for recruitment ensued, with researchers subsequently citing similar enrollment fraud challenges with these new modalities, mirroring those experienced with Web 1.0 and Web 2.0 [12,18-21]. The ongoing pace of change in technology and the continual introduction of new platforms, such as Twitter, Snapchat, TikTok, Threads, and Reddit, have amplified recruitment and enrollment challenges [22,23]. Certainly, the opportunity for potential research participants to misrepresent themselves is not something necessarily unique to the internet and social media [24]; however, the quickly evolving online environment may offer new or enhanced motivations for misrepresentation to obtain an incentive or multiple incentives [25,26] or to deliberately mislead or “troll” researchers [27,28]. Such cases degrade the integrity of the study, erode data integrity, compromise scientific inquiry, and raise difficult ethical questions regarding confidence in results, which in turn may foster distrust and suspicion among truly eligible participants [29-31].

No doubt fostered by the surge in online research during the COVID-19 pandemic, several researchers have recently described methods for assessing fraudulent enrollment [32-35], primarily aimed at postdata collection analysis and algorithms for determining the likelihood of fraud and case exclusion. In this paper, we contribute to this emerging body of research by describing our strategies for detecting and addressing fraud prior to data collection. We present an example from the recruitment of urban American Indian and Alaska Native young women for the formative research phase of a preconceptual health study. Preventing fraudulent data in this context is particularly important. American Indian and Alaska Native communities are often suspicious of and resistant to engagement with research due to a long history of exploitative research practices [36-38]. Indeed, this research exploitation unfolded within the context of centuries of genocidal and assimilationist federal policies designed to destroy American Indian and Alaska Native peoples and their cultures. The resultant generational grief, exacerbated by unscrupulous research practices, has deepened the mistrust of research among both urban- and reservation-based American Indian and Alaska Native communities. This suspicion is a strong call to researchers to rebuild trust [37,39]. Ensuring data authenticity can play a crucial role in helping to bridge this trust between the researcher and the community.

One assimilationist policy, the Indian Relocation Act of 1956, was intended to move American Indian and Alaska Native individuals off reservation lands, out of sovereign Tribal nations, and into urban areas. The result was a concentration of American Indian and Alaska Native individuals in some major US cities, among them Chicago, Los Angeles, Minneapolis, Denver, Phoenix, and Seattle, with gradual spread into suburban and exurban developments in many metropolitan areas. American Indian and Alaska Native individuals living in urban areas—now over 70% of the American Indian and Alaska Native population [40]—do not typically live clustered in neighborhoods. In addition, they are not homogeneous but rather represent rich and diverse cultural backgrounds from their home communities. This dispersion and diversity renders traditional recruitment methodologies ineffective and costly, and thus urban American Indian and Alaska Native individuals have been largely ignored in prior research [41,42]. Yet health disparities are stark and persistent [43-46], with still a sparse research base to inform prevention and treatment [47].

Using the internet and social media for the recruitment of urban American Indian and Alaska Native individuals can provide the opportunity for a broad reach, especially with the growing use of virtual or online engagement via mobile phones [48]. However, using such methodologies could be particularly harmful to American Indian and Alaska Native populations because of the risk of fraudulent cases, as described above. This paper describes the development and integration of Protocol for Increasing Data Integrity in Online Research (PRIOR), which is our 2-step method for preventing fraudulent cases in the recruitment of urban American Indian and Alaska Native young women across multiple internet and social media platforms in our effort to build trust and foreground authentic American Indian and Alaska Native voices in research.

## Methods

### Study Design

We used social media posts and boosted ads plus email flyers to recruit participants nationally for the formative phase of a virtual randomized control trial (RCT), that is, a study design in which recruitment, data collection, and intervention implementation would be administered fully remote through technology. The RCT was designed to test the effectiveness of a culturally tailored mobile health app to prevent alcohol-exposed pregnancy risk among urban American Indian and Alaska Native young women (for details regarding the overall project, see Kaufman et al [49]). We recruited participants for this formative study from February 2019 to August 2020. Finally, the authorship team did not use any generative artificial intelligence in the development of this research protocol nor in the parent RCT study.

### Ethical Considerations

The procedures associated with this study and the RCT, including ethical approval and oversight, have been approved by the Colorado Multiple Institutional Review Board (#18-0574). Additionally, a data safety monitoring board was created to review all protocols associated with the RCT, while also providing oversight on data collection and analyses and the interpretation of results [39]. Informed consent was obtained from all participants, and a US $50 Amazon gift card was issued as compensation for their time. All collected data within this protocol was analyzed for legitimacy and then deidentified for participation in the project. Details about the process are described below*.*

### Participants

To meet eligibility criteria for this formative work, participants had to be female at birth; aged 16-20 years; self-identify as American Indian or Alaska Native; reside within the United States; not live on an American Indian and Alaska Native reservation or village; reside in a city with a population of 50,000 or more; and not be pregnant. Those living in Alaska were excluded since approval for research had not yet been obtained from the relevant research review board.

### Procedures

We used online survey tools to create an eligibility screener. Recruitment ads containing a link to the screener were shared through comprehensive recruitment methods including in-person events and online through email listserves and on social media platforms such as Facebook and Instagram. When in-person events were halted due to the COVID-19 pandemic, all recruitment methods were moved to online platforms. All ads indicated eligible respondents would receive payment.

### Fraud Prevention Integration to Procedures

The screener survey was initially programmed in REDCap (Research Electronic Data Capture; Vanderbilt University) [50], which is a secure web application for building and managing online surveys and databases. Understanding the potential for fraudulent responses, we followed common security guidance [18,51,52] and used reCAPTCHA, a test to distinguish humans from “bots,” or automated algorithms impersonating human engagement [53]. However, despite these security efforts, the screener links were inundated with over 500 fraudulent screener survey responses within 48 hours of launch. Fraudulent responses were quickly identifiable to the research team when the following were identified through analysis of the raw data: (1) continued incorrect responses to survey questions (eg, providing the first and last name as a response to “What is your first name?”); (2) multiple eligible repeated responses from the same town or city; and (3) evidence of trial-and-error responses to eventually achieve eligibility—for example, multiple records with the same contact information, but each record showing incrementally “correct” responses. Additionally, we noticed many of the fraudulent data responses originated from 1 specific recruitment link—Facebook. As soon as this fraudulent activity was detected, we disabled all original links to our screener survey and redistributed recruitment flyers with new links. Even though not all of the recruitment links contained fraudulent data responses, the research team made the difficult decision to redistribute all new links as a precautionary measure. Still, within 2 weeks, we received an additional 1300 fraudulent hits; the research team was only able to discern that 6 participants (0.46% of total responses) were deemed legitimate participants based on self-report. The links were again closed and this time, recruitment efforts were paused to allow time for the development of a more robust protocol to detect and prevent fraudulent attempts at study enrollment.

### Development and Implementation of PRIOR

Subsequently, we developed a 2-step integration verification protocol known as PRIOR. The first step included the addition of four electronic protections to assist in limiting fraudulent hits to the screener as follows: (1) The screener survey was moved to Qualtrics [54] to take advantage of its robust security features, including the option to require a password for survey access or protect against bots using reCAPTCHA. (2) Embedded data points were created to assist in determining fraud. Embedded data points are information automatically collected via survey software from a participant’s device, such as IP address, latitude and longitude, duration (in seconds) of survey use, browser type, and browser version. (3) Timing features were enabled within the survey instrument. This allowed us to require participants to spend a certain amount of time on each screen prior to advancing to the next screen of questions, thus preventing “bots” or program codes from advancing through the survey. (4) Unique URL links were created for each method of recruitment such as each separate social media platform and email.

The second step involved manual verification by phone with prospective participants deemed tentatively eligible based on their screener survey responses. Our phone verification process required tentatively eligible participants to speak by phone with a project team member who asked questions to verify the participant’s eligibility by confirming specific identity information. In the following, we describe each of these steps and their elements in more detail.

### Step 1: Actions to Electronically Detect Fraud

#### Qualtrics Security Features

The antifraudulent features unique to Qualtrics were key to the effectiveness of our new protocol. We first transferred the screener survey to Qualtrics to enable the “prevent ballot box stuffing” feature in addition to reCAPTCHA. The “ballot box stuffing” option in Qualtrics is unique and considered a critical element in mitigating fraud within research [18]. With this feature activated, Qualtrics places a unique browser cookie “marking” the participant’s previous participation within the survey to prevent duplicate entries in the data [55], which in our case, would mitigate trial-and-error attempts to achieve eligibility, as well as multiple fraudulently eligible participants.

#### Geolocation and Embedded Data

Three individual embedded data points—IP address, latitude, and longitude—were used to assess geolocation, as recommended by Ballard et al [18] as a key tool in assisting with mitigating fraud in research. Globally, IP addresses are used to identify specific devices on specific networks, essentially being a unique form of identification in the online sphere [51]. IP addresses in combination with latitude and longitude comparisons create a single variable of geolocation, a critical step as IP addresses have documented inaccuracies as a stand-alone indicator for detecting duplicate responses [56]. This element of fraud prevention is imperfect in cases of legitimately eligible individuals residing in the same household, as devices connected to the same virtual private network will all have the same IP addresses [52]. However, geolocation works more efficiently in tandem with other key embedded data points such as the “Device Identifier” features housed within Qualtrics. This feature enables researchers to collect extra information about the device the participant used, if needed, and attach it to the individual participant’s data [55]. After assessing geolocation, this feature allowed us to compare similarities in “suspicious” cases that appeared fraudulent. This information allowed for a deeper look into the specific circumstances under which the survey was conducted, such as browser-specific information, for example, Google Chrome or Firefox, and the version of the browser used by the participant; and the type of device being used, for example, Android or Apple.

Although these 2 types of variables, geolocation and embedded data points, are separate entities within Qualtrics, they were examined collectively to discern potential fraud. This collective integration occurred in several ways. First, we compared respondent-provided location to geolocation. If the locations differed, the response was flagged as “potentially fraudulent.” Second, device identifiers were examined to determine if there were any cases with several identical responses across respondents with the same browser and browser version. If any of the cases had repeated similar responses, they were flagged as “potentially fraudulent.” All other participants without any sign of potentially fraudulent data were categorized as tentatively eligible.

#### Embedding Timing on Survey Items

We also integrated timing elements into the screener survey. The presence of a “timing” question allows Qualtrics to track the time (in seconds) spent on each page to give the researcher a timestamp variable. This feature also prevents participants from “advancing” within the survey until a certain time has passed [55]. The decision to implement timing on questions within the survey was based on the aforementioned fraudulent data responses the research team received. That is, fraudulent responses were extremely rapid responses that only a bot could provide. To permit timing, we separated screener questions into 3 separate blocks (ie, mobile pages) meaning the eligibility questions did not appear all together but instead on different mobile pages. Doing so allowed the participants to only be able to advance to the next screen after at least 5 seconds had passed and autoadvancing after 60 seconds. The autoadvance feature was included to prevent individuals from memorizing questions and responses and from multiple attempts to respond “correctly.”

#### Unique Recruitment URLs

Unique recruitment screener survey links were created for each separate method of recruitment. More specifically, we created a different link for each of the electronic recruiting methods, such as Facebook, Instagram, and other social media platforms, and we also created individual links for in-person recruitment flyers and for electronic postings via listserves or through email. Creating method-specific recruitment links allowed the research team to “close” a link if rounds of fraudulent responses were occurring without missing potential participants in other sources of recruitment. This was a critical lesson learned by the research team during the analysis of the fraudulent data, where approximately 87.1% of respondents indicated they heard about the study opportunity via Facebook. Had the protocol been in place prior to the onset of data collection, the research team could have isolated the issue to 1 recruitment source. This is critical in ensuring no potential participants are being denied the study opportunity on other platforms.

### Step 2: Verification With Participants

Phone verification was a critical piece of our fraud prevention protocol. Participants deemed tentatively eligible or who had answered all eligibility questions correctly but were flagged as potentially fraudulent via the embedded data and geolocation were contacted by a member of the research team to ascertain eligibility through a series of questions designed to confirm true eligibility. Participants were required to provide a contact number in the screener survey. This number was used to contact participants who were then asked to confirm their birth date, city and state, email address, and how they heard about the study. If participants’ responses affirmed the embedded data points and response they provided in the screener survey, they were enrolled in the study enrollment via an emailed consent form for e-signature; major discrepancies resulted in nonenrollment. For example, 1 “potentially fraudulent” participant, when asked about their residence by a research team member, was told about varying major urban cities by another individual in the background. This major discrepancy resulted in nonenrollment within the study. In contrast, 1 participant listed a major city as their place of residence, and when contacted by a member of the research team, they named a suburb outside of the city. This participant was not disqualified based on this minor discrepancy.

## Results

**Figure 1:**
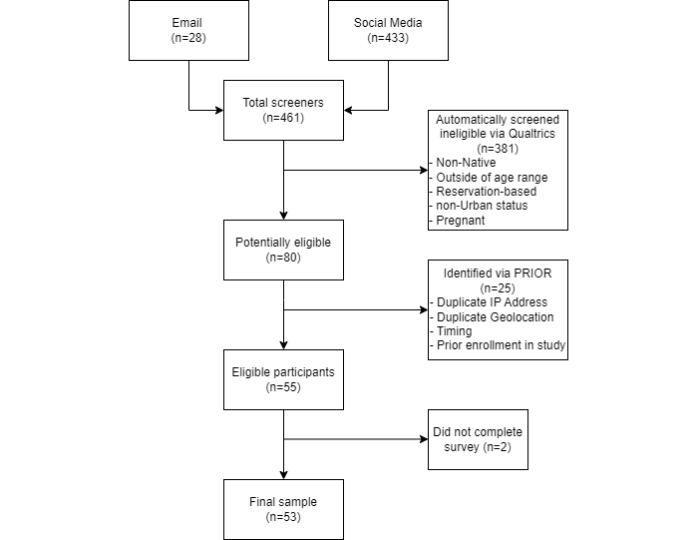
Final participant enrollment after implementation of the PRIOR (Protocol for Increasing Data Integrity in Online Research) protocol.

We relaunched our recruitment campaign with the enhanced fraud protection protocol on May 11, 2020. After the implementation of this enhanced PRIOR protocol, we had 461 individuals total come through the combined screener surveys across all platforms (Figure 1)—433 total individuals for social media (Facebook, Instagram, and Twitter), and 28 individuals for email-based recruitment. A total of 80 participants were deemed tentatively eligible, and of these 80, a total of 55 (69%) participants met the inclusion criteria and were verified as unique, and 2 participants did not complete the survey, resulting in a final enrollment of 53 participants for the formative study. Of the remaining 25 (31%) participants that were not eligible, 6 participants were excluded because they did not live in a city meeting the population size criterion, and 4 participants attempted to re-enroll in the study after receiving their participant compensation, an action that was also flagged by our protocol. The remaining 15 participants were identified as fraudulent responses based on the protocol set in place.

Before the implementation of PRIOR, fraudulent hits would begin with the first initial response well exceeding 5000 seconds (or about 83 min), which was far too long for a short set of screener questions, followed by several bouts of all eligible responses completed in less than 30 seconds. After the implementation of the enhanced fraud prevention protocol, the research team was able to determine that, on average, participants took 105.07 seconds to successfully complete the screener survey’s 9 questions.

In the final sample (n=53), 4 participants were within the same geolocation—one set of siblings and one set of cousins residing in the same household. In these cases of family relations, only 2 individual responses had similar geolocation data points and device identifiers. In the follow-up phone call to the potential participants, we learned that 1 sibling informed the other of the research opportunity. Comparatively, in cases marked as fraudulent, there were more than 20 individual responses with differing geolocation data points, but device identifiers, such as browser version and operating systems, remained consistent among these individual responses. Finally, via the embedded data points, we identified some participants who did not successfully screen into the study and tried again with different responses to meet eligibility criteria. These 4 participants were excluded from the study and labeled as fraudulent.

## Discussion

### Principal Findings

As online data collection in diverse environments increases, so too increases the opportunities for—and experiences that researchers will have with—fraud, either perpetuated by bots or individuals with malintent. As we demonstrate in this work, the methods to detect and prevent fraudulent participation in online surveys that have been endorsed to date were insufficient and, thus, ineffective when attempting to recruit and enroll the sample for this study. The 2-step strategy described here contributes to a small but growing literature to address fraud in survey research [32,57] and reduce this threat to research integrity.

Our PRIOR protocol is comprised of many small points of information; however, these points, when reviewed together, created a full picture of the legitimacy of each participant and proved to be a powerful tool to determine and detect fraud. Changing data collection platforms, and using commonly recommended features of Qualtrics such as reCAPTCHA and “ballot box stuffing” prevention, was clearly impactful in the initial screening of potential participants. Of those who passed this initial stage, our added checks identified another 31% who were not eligible. Although this process is labor-intensive, it helped protect the integrity of the data. This is especially crucial for individuals working with marginalized populations, such as the American Indian and Alaska Native population, who have been subject to research harm in the past [58]. Further, during the phone verification, the research team heard from many participants that they appreciated the extra security measures in place and felt more comfortable enrolling in a virtual research project having talked with a study team member first. The research team hopes this critical step can aid in re-establishing trust between academia and American Indian and Alaska Native communities by making research teams more personable to the participants and actively showing that protections are in place for data collected in any phase of the study—from enrollment to participation in the study itself.

Having a robust security protocol in place helps researchers discern topics of relevance for communities and populations and adds credibility to the collected and analyzed data. Researchers who use this method can improve their reach and legitimacy for research with hidden or hard-to-reach populations and prevent fraudsters from disparaging the integrity of the overall data and receiving compensation inappropriately. Further, integrating a fraudulent data detection protocol into any research endeavor with American Indian and Alaska Native communities or other hard-to-reach populations helps build a foundation within the literature, which simultaneously ensures that data collection is ethical and reflects the authentic views of the community. Additionally, we caution researchers to limit how much information is relayed online to potential participants regarding respondent compensation, especially if research teams plan to use social media accounts to “boost” their recruitment links as ads. Doing so can attract a larger number of fraudulent respondents trying to access immediate funds via respondent compensation. While we note that such steps in a fraudulent data protocol, such as PRIOR, are time and labor-intensive, taking these steps will help to bridge the gap of research distrust and provide the community with accurate and authentic data.

### Limitations

While there are many strengths found within this protocol, several potential limitations should be considered when using PRIOR. First, there are ethical concerns regarding the collection of both IP addresses and geolocation variables. Researchers must ensure that a plan is in place to delink these data points from participant responses to surveys or other data collection materials and to permanently destroy these variables after verification of identity has occurred. As an example, our research team immediately delinks all collected IP addresses from future participant responses and deletes all related variables. Additionally, the collected IP addresses are limited to only specific members of the research team and are kept in password-protected filing systems. Finally, the rigor involved in verifying authentic participants may pose too great a barrier for some eligible participants who may not complete the process. As noted, we found that most participants were pleased with the safeguards in place. While seemingly costly, the benefits include authentic participation, data integrity, and minimal losses in faulty respondent compensation.

### Conclusions

Fraudulent surveys compromise the authenticity and integrity of the data, as well as deplete scarce research resources including respondent compensation and personnel time. It is crucial for researchers working with American Indian and Alaska Native communities or other populations that have experienced research harm to guard against online misrepresentations. Such protocols are critical to protecting the integrity of the data—that is, to ensure that data collected represent authentic voices of the population, in this case, urban American Indian and Alaska Native young women. Ensuring the integrity of the data in this way promotes trust between researchers and vulnerable or previously exploited communities and fosters the identification of authentic needs and priorities of the populations involved with the research. As new technologies, for example, machine learning or artificial intelligence, are used in virtual research methodologies, modification of, or new developments in, existing protective protocols will be essential. The PRIOR protocol described here advances an approach that may serve to prevent fraudulent survey participation in the short term and to foster new and expanded applications concurrent with new data collection technologies.

## Abbreviations

PRIOR: Protocol for Increasing Data Integrity in Online Research
RCT: randomized control trial
REDCap: Research Electronic Data Capture

## Data Availability

Study data were provided by individuals residing in urban American Indian communities in the United States. We respect the rights of American Indian, Alaska Native, Native Hawaiian, and other Indigenous groups to protect research data that pertains to their communities. Questions about data access may be directed to the Centers for American Indian and Alaska Native Health Data Access Committee (DAC). Please contact Ms Sara Mumby, DAC Coordinator, Centers for American Indian and Alaska Native Health, Colorado School of Public Health, University of Colorado Anschutz Medical Campus at sara.mumby@cuanschutz.edu.

## References

[1] Solanki MR, Dongaonkar A (2016). A journey of human comfort: web 1.0 to web 4.0. Int J Res Sci Innov.

[2] Etter JF, Perneger TV (2001). A comparison of cigarette smokers recruited through the internet or by mail. Int J Epidemiol.

[3] Mathew M, Morrow JR, Frierson GM, Bain TM (2011). Assessing digital literacy in web-based physical activity surveillance: the WIN study. Am J Health Promot.

[4] Bull SS, Lloyd L, Rietmeijer C, McFarlane M (2004). Recruitment and retention of an online sample for an HIV prevention intervention targeting men who have sex with men: the smart sex quest project. AIDS Care.

[5] Rhodes SD, Bowie DA, Hergenrather KC (2003). Collecting behavioural data using the world wide web: considerations for researchers. J Epidemiol Community Health.

[6] Hildebrand J, Burns S, Zhao Y, Lobo R, Howat P, Allsop S, Maycock B (2015). Potential and challenges in collecting social and behavioral data on adolescent alcohol norms: comparing respondent-driven sampling and web-based respondent-driven sampling. J Med Internet Res.

[7] Sadler AG, Mengeling MA, Torner JC, Smith JL, Franciscus CL, Erschens HJ, Booth BM (2013). Feasibility and desirability of web-based mental health screening and individualized education for female OEF/OIF reserve and national guard war veterans. J Trauma Stress.

[8] Buller DB, Meenan R, Severson H, Halperin A, Edwards E, Magnusson B (2012). Comparison of 4 recruiting strategies in a smoking cessation trial. Am J Health Behav.

[9] Bull SS, Vallejos D, Levine D, Ortiz C (2008). Improving recruitment and retention for an online randomized controlled trial: experience from the Youthnet study. AIDS Care.

[10] Gordon JS, Akers L, Severson HH, Danaher BG, Boles SM (2006). Successful participant recruitment strategies for an online smokeless tobacco cessation program. Nicotine Tob Res.

[11] McClure JB, Greene SM, Wiese C, Johnson KE, Alexander G, Strecher V (2006). Interest in an online smoking cessation program and effective recruitment strategies: results from project quit. J Med Internet Res.

[12] Pequegnat W, Rosser BRS, Bowen AM, Bull SS, DiClemente RJ, Bockting WO, Elford J, Fishbein M, Gurak L, Horvath K, Konstan J, Noar SM, Ross MW, Sherr L, Spiegel D, Zimmerman R (2007). Conducting internet-based HIV/STD prevention survey research: considerations in design and evaluation. AIDS Behav.

[13] Fortune T, Wright E, Juzang I, Bull S (2010). Recruitment, enrollment and retention of young black men for HIV prevention research: experiences from the 411 for safe text project. Contemp Clin Trials.

[14] Jenkins RA (2012). Recruiting substance-using men who have sex with men into HIV prevention research: current status and future directions. AIDS Behav.

[15] Temple EC, Brown RF (2011). A comparison of internet-based participant recruitment methods: engaging the hidden population of cannabis users in research. J Res Pract.

[16] Web 2.0. Wikipedia.

[17] Hendricks D (2024). Complete history of social media: then and now. Small Business Trends.

[18] Ballard AM, Cardwell T, Young AM (2019). Fraud detection protocol for web-based research among men who have sex with men: development and descriptive evaluation. JMIR Public Health Surveill.

[19] Ballard AM, Cooper HL, Young AM (2019). Web-based eligibility quizzes to verify opioid use and county residence among rural young adults: eligibility screening results from a feasibility study. JMIR Res Protoc.

[20] Marshall BDL, Green TC, Elston B, Yedinak JL, Hadland SE, Clark MA (2018). The effectiveness of internet- and field-based methods to recruit young adults who use prescription opioids nonmedically. Subst Use Misuse.

[21] Gazmararian JA, Yang B, Elon L, Graham M, Parker R (2012). Successful enrollment in Text4Baby more likely with higher health literacy. J Health Commun.

[22] Ford KL, Albritton T, Dunn TA, Crawford K, Neuwirth J, Bull S (2019). Youth study recruitment using paid advertising on Instagram, Snapchat, and Facebook: cross-sectional survey study. JMIR Public Health Surveill.

[23] Pozzar R, Hammer MJ, Underhill-Blazey M, Wright AA, Tulsky JA, Hong F, Gundersen DA, Berry DL (2020). Threats of bots and other bad actors to data quality following research participant recruitment through social media: cross-sectional questionnaire. J Med Internet Res.

[24] Gupta A (2013). Fraud and misconduct in clinical research: a concern. Perspect Clin Res.

[25] Konstan JA, Rosser BRS, Ross MW, Stanton J, Edwards WM (2005). The story of subject naught: a cautionary but optimistic tale of internet survey research. J Comput-Mediat Comm.

[26] Kramer J, Rubin A, Coster W, Helmuth E, Hermos J, Rosenbloom D, Moed R, Dooley M, Kao YC, Liljenquist K, Brief D, Enggasser J, Keane T, Roy M, Lachowicz M (2014). Strategies to address participant misrepresentation for eligibility in web-based research. Int J Methods Psychiatr Res.

[27] Bauermeister JA, Pingel E, Zimmerman M, Couper M, Carballo-Diéguez A, Strecher VJ (2012). Data quality in web-based HIV/AIDS research: handling invalid and suspicious data. Field methods.

[28] Chandler J, Sisso I, Shapiro D (2020). Participant carelessness and fraud: consequences for clinical research and potential solutions. J Abnorm Psychol.

[29] Kosinski M, Matz SC, Gosling SD, Popov V, Stillwell D (2015). Facebook as a research tool for the social sciences: opportunities, challenges, ethical considerations, and practical guidelines. Am Psychol.

[30] Teitcher JEF, Bockting WO, Bauermeister JA, Hoefer CJ, Miner MH, Klitzman RL (2015). Detecting, preventing, and responding to "fraudsters" in internet research: ethics and tradeoffs. J Law Med Ethics.

[31] Griffin M, Martino RJ, LoSchiavo C, Comer-Carruthers C, Krause KD, Stults CB, Halkitis PN (2022). Ensuring survey research data integrity in the era of internet bots. Qual Quant.

[32] Storozuk A, Ashley M, Delage V, Maloney EA (2020). Got bots? practical recommendations to protect online survey data from bot attacks. TQMP.

[33] Lawlor J, Thomas C, Guhin AT, Kenyon K, Lerner MD, Drahota A (2021). Suspicious and fraudulent online survey participation: introducing the REAL framework. Methodol Innov.

[34] Goodrich B, Fenton M, Penn J, Bovay J, Mountain T (2023). Battling bots: Experiences and strategies to mitigate fraudulent responses in online surveys. Appl Eco Perspect Pol.

[35] Wang J, Calderon G, Hager ER, Edwards LV, Berry AA, Liu Y, Dinh J, Summers AC, Connor KA, Collins ME, Prichett L, Marshall BR, Johnson SB (2023). Identifying and preventing fraudulent responses in online public health surveys: lessons learned during the COVID-19 pandemic. PLOS Glob Public Health.

[36] Walters KL, Johnson-Jennings M, Stroud S, Rasmus S, Charles B, John S, Allen J, Kaholokula JK, Look MA, de Silva M, Lowe J, Baldwin JA, Lawrence G, Brooks J, Noonan CW, Belcourt A, Quintana E, Semmens EO, Boulafentis J (2020). Growing from our roots: strategies for developing culturally grounded health promotion interventions in American Indian, Alaska Native, and Native Hawaiian communities. Prev Sci.

[37] Walters KL, Walls ML, Dillard DA, Kaur JS (2019). American Indian and Alaska Native research in the health sciences: critical considerations for the review of research applications. Tribal Health Research Office (THRO), National Institutes of Health.

[38] Sterling RL (2011). Genetic research among the Havasupai—a cautionary tale. Virtual Mentor.

[39] Reed ND, Sarche M, Shrestha U, Bull S, Howley CT, Shangreau C, Asdigian NL, Vossberg RL, Leon JS, Kaufman CE (2023). Creating a virtual indigenist community-based participatory approach: lessons learned from centering urban native young women in research. Adv Res Sci.

[40] 2021: ACS 5-Year estimates selected population detailed tables (B011003). urban-rural estimates, American Indian and Alaska Native alone or in combination with one or more races. U.S. Census Bureau.

[41] Yuan NP, Bartgis J, Demers D (2014). Promoting ethical research with American Indian and Alaska native people living in urban areas. Am J Public Health.

[42] James RD, West KM, Claw KG, EchoHawk A, Dodge L, Dominguez A, Taualii M, Forquera R, Thummel K, Burke W (2018). Responsible research with urban American Indians and Alaska Natives. Am J Public Health.

[43] Jones DS (2006). The persistence of American Indian health disparities. Am J Public Health.

[44] Kruse G, Lopez-Carmen VA, Jensen A, Hardie L, Sequist TD (2022). The Indian Health Service and American Indian/Alaska Native Health outcomes. Annu Rev Public Health.

[45] Melkonian SC, Jim MA, Haverkamp D, Wiggins CL, McCollum J, White MC, Kaur JS, Espey DK (2019). Disparities in cancer incidence and trends among American Indians and Alaska natives in the United States, 2010-2015. Cancer Epidemiol Biomarkers Prev.

[46] Tapper EB, Parikh ND (2018). Mortality due to cirrhosis and liver cancer in the United States, 1999-2016: observational study. BMJ.

[47] Intervention research to improve Native American Health (PAR-20-238). National Institutes of Health.

[48] Reed ND, Peterson R, Ghost Dog T, Kaufman CE, Kelley A, Craig Rushing S (2022). Centering native youths' needs and priorities: findings from the 2020 native youth health tech survey. Am Indian Alsk Native Ment Health Res.

[49] Kaufman CE, Asdigian NL, Reed ND, Shrestha U, Bull S, Begay RL, Shangreau C, Howley CT, Vossberg RL, Sarche M (2023). A virtual randomized controlled trial of an alcohol-exposed pregnancy prevention mobile app with urban American Indian and Alaska native young women: native WYSE CHOICES rationale, design, and methods. Contemp Clin Trials.

[50] Harris PA, Taylor R, Thielke R, Payne J, Gonzalez N, Conde JG (2009). Research Electronic Data Capture (REDCap)—a metadata-driven methodology and workflow process for providing translational research informatics support. J Biomed Inform.

[51] Ball HL (2019). Conducting online surveys. J Hum Lact.

[52] Zhang L (2008). A retrospective view of network address translation. IEEE Network.

[53] von Ahn L, Maurer B, McMillen C, Abraham D, Blum M (2008). reCAPTCHA: Human-based character recognition via web security measures. Science.

[54] (2023). Qualtrics XM (2019-2020).

[55] Snow J (2011). The complete research suite: a step-by-step guide to using qualtrics. Qualtrics.

[56] Poese I, Uhlig S, Kaafar MA, Donnet B, Gueye B (2011). IP geolocation databases: unreliable?. SIGCOMM Comput Commun Rev.

[57] Xu Y, Pace S, Kim J, Iachini A, Bailey King L, Harrison T, DeHart D, Levkoff SE, Browne TA, Lewis AA, Kunz GM, Reitmeier M, Utter RK, Simone M (2022). Threats to online surveys: recognizing, detecting, and preventing survey bots. Social Work Research.

[58] Gone JP (2023). Researching with American Indian and Alaska native communities: pursuing partnerships for psychological inquiry in service to indigenous futurity. APA Handbook of Research Methods in Psychology: Research Designs: Quantitative, Qualitative, Neuropsychological, and Biological.

